# Psoriasis and Myasthenia Gravis: A Common Th-17 Pathway

**DOI:** 10.7759/cureus.23090

**Published:** 2022-03-12

**Authors:** Fouad El Sayed, Mariam Kabbani

**Affiliations:** 1 Dermatology, Lebanese University Faculty of Medicine, Beirut, LBN

**Keywords:** psoriasis treatment, il-17 blockers, ixekizumab, myasthenia gravis, psoriasis

## Abstract

Psoriasis is a chronic inflammatory skin disease whose treatment arsenal is expanding by the day. However, when comorbidities coexist, therapy can be challenging. We report a case of a 55-year-old female with steroid-dependent myasthenia gravis who presented with a severe form of chronic plaque psoriasis. After the failure of topical corticosteroids and phototherapy, the patient was started on ixekizumab. This anti-IL-17 antibody led not only to the clearance of the psoriatic lesions but also to the remission of the myasthenic symptoms. While on this medication, the patient was able to taper down and discontinue the oral corticosteroids. The remission of the symptoms of myasthenia gravis during this treatment supports the role of IL-17 cytokines in the pathogenesis of this disease and adds it as a management option in steroid-dependent cases.

## Introduction

Psoriasis is a common chronic immune-mediated disease presenting most commonly (80%) with well-defined scaly erythematous plaques [1]. On the other hand, myasthenia gravis is a relatively uncommon condition with a prevalence of 150-250 cases per million. It is a chronic autoimmune disorder characterized by fatigability and weakness of skeletal muscles [2]. The coexistence of these two diseases is very rare with only five cases reported. Moreover, the pathogenesis of psoriasis results from the interplay of different cytokines wherein the role of the IL-23/IL-17 axis is well established [3]. Nonetheless, the role of IL-17 in the pathogenesis of myasthenia gravis is newly discovered and remains under investigation [4]. To our knowledge, no case is reported in the literature describing the use of anti-IL-17 antibodies in the management of myasthenia gravis. Herein, we report a rare case of psoriasis associated with myasthenia gravis wherein the IL-17 pathway was targeted to manage both diseases.

## Case presentation

**Figure 1:**
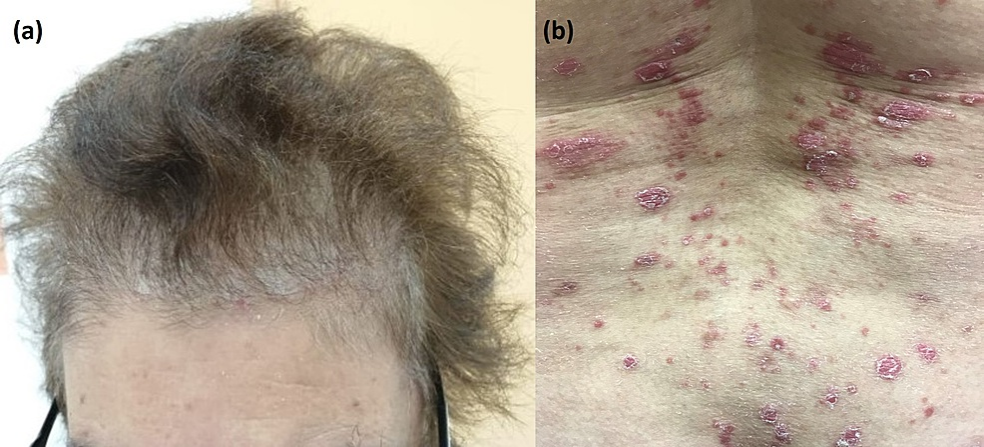
Scaly erythematous plaques of psoriasis located on (a) the scalp and (b) the trunk.

A 55-year-old female with a five-year history of myasthenia gravis presented for recent exacerbation of her psoriasis which was diagnosed at the age of 20. She is obese with a BMI of 30.84 kg/m^2^ and has developed non alcoholic fatty liver disease (NAFLD). Moreover, the patient had been suffering from refractory myasthenia gravis for which she received, one year prior to presentation, two intravenous courses of Rituximab six months apart. Since then, the patient had been taking prednisone 10 mg daily for the management of her myasthenic symptoms. On examination, we noted diffuse erythematous scaly plaques (Figures 1a, 1b) covering 41% of her body surface area (PASI: 16) and nail involvement but without arthritis.

**Figure 2:**
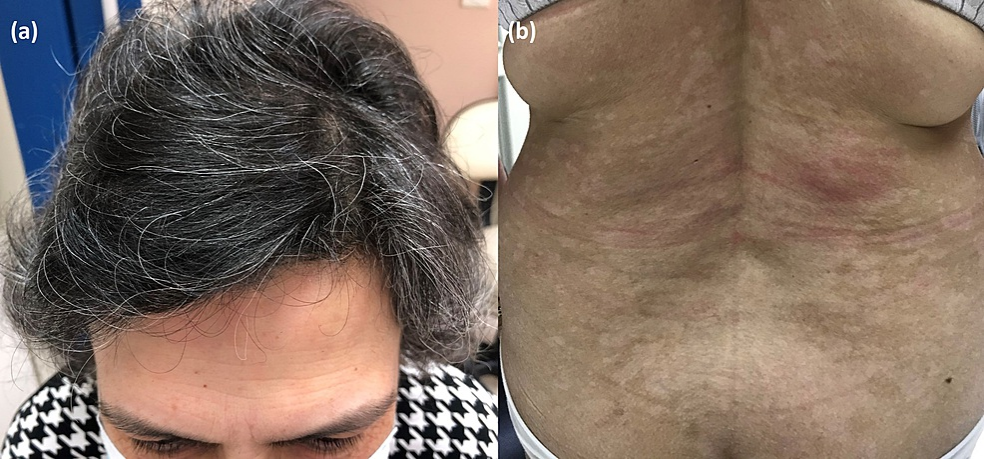
Resolution of psoriatic lesions after four months of ixekizumab therapy with (a) hair regrowth and (b) post-inflammatory hypopigmentation of the trunk.

We started her on topical corticosteroids and narrowband UVB-311nm phototherapy (NBUVB). Nonetheless, after 15 sessions of NBUVB, the patient did not show any response and phototherapy was stopped. Since acitretin is unavailable in Lebanon and methotrexate is incompatible with her NAFLD, pre-cyclosporine screening revealed a high blood pressure of 172 mmHg/101 mmHg. Therefore, we considered treatment with a biologic agent, and after consultation with her neurologist, she was started on ixekizumab injections at a dose of 160 mg at week zero, followed by 80 mg every two weeks for three months, then 80 mg every four weeks. One month into the therapy, she was able to successfully taper off the prednisone, and after four months, she remained off oral corticosteroids without any myasthenic symptoms and with marked improvement (Figures 2a, 2b) of her psoriatic lesions (PASI 90).

However, due to the subsequent unavailability of ixekizumab for two months, the patient suffered from a severe relapse of her myasthenia gravis during the second month. She was hospitalized, received five sessions of plasmapheresis and IVIG, and then resumed Rituximab injections with daily prednisone 30 mg. No psoriasis relapse was observed. As soon as the medication was again available the next month, the injections of ixekizumab were restarted with rapid tapering of the oral corticosteroids.

## Discussion

Myasthenia gravis is a chronic autoimmune disorder in which the clinical presentation is dominated by fatigability and weakness of skeletal muscles [2]. In 85% of cases, there exist autoantibodies against acetylcholine receptors (ACR) wherein plasma cells and activated T cells may play a role in the inflammation at the neuromuscular junction and the induction of these autoantibodies [2,5]. Although myasthenia gravis can be associated with other autoimmune disorders in 15% of the cases namely thyroiditis, systemic lupus erythematosus, and rheumatoid arthritis [2], psoriasis rarely coexists with myasthenia gravis. To our knowledge, only five cases (Table 1) are reported in the literature describing an association between myasthenia gravis and psoriasis [6-8]. Including our patient, all cases were anti-ACR positive, and four out of six patients were females, which can be explained by the female predominance of myasthenia gravis [2]. Psoriasis preceded myasthenia gravis in only two cases, and all, apart from our case, were treated by topical corticosteroids either alone or in combination with phototherapy [6-8]. 

**Table 1:** Reported associations of psoriasis and myasthenia gravis. +: present; -: not present; ACR: anti-acetylcholine receptors antibodies; AOO: age of onset; F; female; M; male; MG: myasthenia gravis; NBUVB: Narrowband ultraviolet B therapy; NM: not mentioned; PASI: Psoriasis area severity index; PsA: psoriatic arthritis; PsO: psoriasis; PUVA: Psoralen ultraviolet A therapy; TCS: Topical corticosteroids; VD3: Vitamin D3.

Cases	Sex	Age	Preexisting disease	ACR	MG treatment	PsO treatment	PsA	PASI on presentation
Kwan et al, [6]	M	36	MG	+	NM	NM	NM	NM
Koc et al. [7]	F	53	MG	+	Thymectomy Pyridostigmine	Methotrexate TCS and vaseline ointment	+	NM
Takahashi et al. [8], Patient 1	F	37	MG	+	Thymectomy Prednisolone 4.5 mg Tacrolimus 3mg	PUVA TCS and VD3 ointment	NM	16.1
Takahashi et al. [8], Patient 2	F	56	PsO	+	Thymectomy Prednisolone 10mg Tacrolimus 3mg	TCS and VD3 ointment	NM	NM
Takahashi et al. [8], Patient 3	M	47	MG	+	Thymectomy Prednisolone 2.5mg Tacrolimus 3 mg	TCS and VD3 ointment	NM	9.8
Our patient	F	55	PsO	+	Thymectomy Rituximab Prednisone 10mg	TCS NBUVB Ixekizumab	-	16

The pathogenesis of psoriasis results from the secretion of IFN-α by plasmacytoid dendritic cells (pDC) in response to nucleic acids released from damaged keratinocytes. This leads to the maturation of conventional dendritic cells (cDC) which then release IL-12, IL-23 and TNF-α. These cytokines promote the differentiation of naïve T cells into Th1, Th22, and Th17 cells [3]. IL-17, mainly secreted by Th17 cells, causes abnormal proliferation of keratinocytes and stimulates them to release various chemokines [9], thereby resulting in the formation of classic psoriatic plaques. However, recent articles suggest a role of Th17 cells in anti-ACR-positive myasthenia gravis [5], The suggested mechanism of thymic inflammation involves dysregulation of interferon type I pathway which leads to overexpression of IL-23 and thus to overproduction of IL-17 [10]. The pivotal role of the IL-23/IL-17 axis is further supported by our findings of attaining remission while on anti-IL-17 antibody and experiencing a relapse after this therapy was discontinued.

The mainstay of treatment of myasthenia gravis is the use of symptomatic treatment and immunosuppression. The former, which most importantly include acetylcholinesterase inhibitors, are used mainly in mild cases with localized symptoms as a maintenance treatment. For more severe cases, the use of immunosuppression is critical including corticosteroids and corticosteroid-sparing drugs such as mycophenolate mofetil, cyclosporin, and rituximab [11]. As for psoriasis, mild presentations can be treated with topical treatments, mainly corticosteroids, analogs of vitamin D, and calcineurin inhibitors. However, moderate-to-severe psoriasis often requires systemic treatment. When solely present, psoriasis can be treated with numerous medications ranging from conventional oral systemic drugs, such as methotrexate, to a wide range of biologic therapies targeting TNF-a, IL-17, or IL-23 pathways [1]. However, treatment proves to be challenging when comorbidities and autoimmune diseases coexist.

## Conclusions

We report the first case describing the efficacy of anti-IL-17 antibodies in the management of both myasthenia gravis and psoriasis. This supports the role of Th-17 cells in the pathogenesis of myasthenia gravis, known key players in the pathophysiology of psoriasis, and demonstrates the possible role of anti-IL-17 antibodies in the treatment of myasthenia gravis. Therefore, randomized trials are needed to further study the effect of these antibodies in the management of steroid-dependent myasthenia gravis.
